# Quick Analysis of Organic Amendments via Portable X-ray Fluorescence Spectrometry

**DOI:** 10.3390/ijerph16224317

**Published:** 2019-11-06

**Authors:** Rafael López-Núñez, Fátima Ajmal-Poley, José A. González-Pérez, Miguel Angel Bello-López, Pilar Burgos-Doménech

**Affiliations:** 1 Instituto de Recursos Naturales y Agrobiología de Sevilla (IRNAS-CSIC), Avda. Reina Mercedes 10, 41012 Sevilla, Spain; fatima.ap94@gmail.com (F.A.-P.); jag@irnase.csic.es (J.A.G.-P.); pburgos@irnase.csic.es (P.B.-D.); 2 Department of Analytical Chemistry, Faculty of Chemistry, Universidad de Sevilla, c/Prof. García González, s/n, 41012 Sevilla, Spain; mabello@us.es

**Keywords:** compost, sewage sludge, *aqua regia*-soluble content, heavy metal, urban garden, loss on ignition, organic matter determination, multiple linear regression

## Abstract

The determination of heavy metals in soils and organic amendments, such as compost, manure, biofertilizer, and sludge, generally involves the digestion of samples with *aqua regia*, and the determination of those in the solution using various techniques. Portable X-ray fluorescence (PXRF) has many advantages in relation to traditional analytical techniques. However, PXRF determines the total elemental content and, until now, its use for the analysis of organic amendments has been limited. The objective of this work is the calibration of a PXRF instrument to determine the *aqua regia*-soluble elemental contents directly in solid samples of organic amendments. Our proposal will avoid the digestion step and the use of other laboratory techniques. Using a training set of samples, calibration functions were obtained that allow the determination of the *aqua regia*-soluble contents from the PXRF readings of total contents. The calibration functions (obtained by multiple linear regression) allowed the quantitative determination of the *aqua regia*-soluble contents of Fe, K, P, S, Zn, Cu, Pb, Sr, Cr, and Mn, as well as the organic matter content and a semi-quantitative assessment of Al, Ca, V, Ba, Ni, and As contents. The readings of Si, Fe, Al, Ca, K, or S were used as correction factors, indicating that the calibrations functions found are truly based on the chemical composition of the sample matrix. This study will allow a fast, cheap, and reliable field analysis of organic amendments and of other biomass-based materials.

## 1. Introduction

Urban agriculture is a growing activity in many cities on a worldwide scale [1,2]. Urban agriculture is practised in both developed and developing economies, although it can serve different purposes in different places, such as recreation or food security [3]. Under both approaches, there is a revitalised interest in research concerning urban agriculture from a variety of perspectives, including landscaping and urban planning [4], social and economic aspects [5], health and the environment [6], as well as technical subjects [7].

There is debate over the advantages and disadvantages of urban agriculture. There are many well-documented benefits of urban agriculture: subsistence survival, food sovereignty and security, reduced food transportation distance, carbon sequestration, a potentially reduced urban heat island effect, improved physical and mental health, improved aesthetics, community building, employment opportunities, improved local land prices, shortened supply chains, and, thus, reduced price differentials between producers and consumers, provision of habitats for wildlife, and waste recycling [8].

A major concern of urban agriculture lies in risks related to the eventual pollution of urban products. Fruits and vegetables grown in cities could be polluted by undesirable organic or inorganic substances [2], and there are several studies that have focused on trace elements found in urban fruits and vegetables. Metals can reach vegetables through different pathways, one of them being the use of polluted fertilizers, composts, sewage sludge, and livestock manure [9]. The reviews of Alloway [9] and Wei and Yang [10] reported on anthropogenic sources of metal contamination.

The determination of heavy metals in soils and organic amendments generally involves the digestion of samples with *aqua regia*, and the determination of those in the solution using various analytical techniques such as ICP-OES, atomic absorption, and others. Urban gardeners generally lack the financial and technical requirements necessary to test metal contamination in their soils and amendments using these analytical techniques.

Recently, improvements in X-ray tube and detector technologies have resulted in field-portable instruments, such as portable X-ray fluorescence (PXRF), that enable such uses as geologic investigations [11], as well as having environmental, pedological, and agronomic applications [12]. PXRF has many advantages in relation to traditional analytical techniques and is less expensive. PXRF analysis is non-destructive and very fast, with a simple or no sample treatment. The instrument could be used in the laboratory or in the field. The technique has a wide range of quantifications, having detection limits poorer than those of large laboratory-based XRF instruments or of ICP techniques, but it is sufficient for environmental monitoring of a large number of elements that can be determined simultaneously [12,13]. Despite lower detection limits of the chemical methods, sometimes these become more imprecise and inaccurate than XRF spectrometry, mainly due to the fact that the latter requires virtually no sample pretreatment [14]. There are approved protocols for using this instrument in soils and sediments [15,16]. Recently, Caporale et al. [17] evaluated the possibility of assessing the *aqua regia*-extractable content of metals from a multi-element profile of the soil using a PXRF analyser. These authors indicated that the discrepancy between the ‘pseudototal’ metal contents measured in soil samples by *aqua regia* and the ‘total’ contents measured by PXRF strongly depended on the properties of the element to be analysed, the chemical and mineralogical composition of the soil matrix, the organic matter content as well as of the type of acid used in the total extraction. Sharma et al. [18] applied multiple linear regression to a modeling dataset to establish the relationship between lab-determined soil Cation Exchange Capacity and eight variables from PXRF elemental data. They further improved the model using auxiliary input data (soil clay, pH, organic matter) as modeling variables; when using only clay, organic matter, and two elements (Cr, Ni) to make the prediction they obtained better regression coefficients.

Despite the possibilities of this technique, until now its use for the analysis of compost and organic amendments has been limited to a few investigations [19,20,21,22,23,24], and the possibility for quantitative determination has been limited to a few elements. McWhirt et al. [22] and Havukainen et al. [23] found only eight elements that could be quantitatively determined in dry samples, and only qualitative results were obtained for a few more. They indicated that both the physical and the chemical properties of the samples affected measurement accuracy; significant influences due to moisture content, particle size, sample heterogeneity, positioning of samples, matrix effects (e.g., inter-element effects), calibration and reference standards, sample pre-treatment (e.g., drying, sieving, and homogenization), and the organic content of the sample were found.

Nevertheless, these studies concluded that there is a great potential for widespread evaluation and definitive quantification of elemental concentrations, and that the lower cost, faster speed, and the portability of PXRF may outweigh the slight loss of accuracy, especially when the field evaluation of composted materials is a primary objective.

We hypothesized that elemental data obtained from PXRF analyser can serve as a proxy for *aqua regia*-soluble contents of organic amendments. We also hypothesized that *aqua regia*-soluble contents could be reliably determined using multiple linear regression including correction terms based on the contents of major elements, such as Si and Fe. The objective of this work is the calibration of a PXRF instrument to determine the *aqua regia*-soluble elemental contents, directly from solid samples of organic amendments, avoiding previous digestion processes and the use of other laboratory techniques. Using a training set of samples, calibration functions based on multiple linear regression will be obtained that could allow the determination of the *aqua regia*-soluble contents from the PXRF readings of the total contents.

To the authors’ knowledge, such an approach has not been used to date. Not even in the case of soils, in which the relationship between PXRF and *aqua regia*-extractable contents has been more studied, has this correction been proposed, despite Si being a frequent element in soils.

## 2. Materials and Methods

### 2.1. Field Portable X-ray Fluorescence (PXRF) Analysis

The analyzer Niton XL3t 950s GOLDD + XRF (Thermo Scientific Inc., Billerica, MA, USA), with its laboratory stand, was used in this study. The analyzer was equipped with an X-ray tube with an Ag anode, operated at 50 keV, 200 µA, and 2 W, with a geometrically optimized large Si drift detector (GOLDD). The analyzer can measure a range of elements, from Mg to U. Two factory calibration modes, known as the Mining (M) mode and the Soils (S) mode, were used for the measurements. In the M mode the following elements could be detected: Ba, Sn, Cd, Pd, Ag, Mo, Nb, Zr, Sr, Bi, As, Se, Hg, W, Pb, Zn, Cu, Ni, Co, Fe, Mn, Cr, V, Ti, Ca, Cl, K, S, P, Si, and Al. The S mode is recommended for soil metallic elements at low concentrations, and the following elements could be detected: Ba, Sb, Sn, Cd, Ag, Mo, Zr, Sr, U, Rb, Th, Pb, Se, As, Tl, Hg, Zn, Cu, Ni, Co, Fe, Mn, Cr, V, Ti, Ca, K, Cl, S, and P.

Dried and finely ground samples were measured by filling an XRF container (model SC-4331, 26 mm internal diameter, 24 mm height, Premier Lab Supply Inc., Port St. Lucie, FL, USA) capped with a 4 μm propylene film (model 240–255, 63 mm diameter, Premier Lab Supply Inc., Port St. Lucie, FL, USA). The samples were scanned in triplicate in each of the M and S modes and the average elemental content for each mode used hereafter. The analysis time for each scan was 90 s for the S mode and 120 s for the M mode.

### 2.2. Samples

The samples of organic amendments corresponded to the MARSEP program, which is part of the Wageningen Evaluating Programmes for Analytical Laboratories (WEPAL) [25]. Samples corresponding to the quarterly periods one and three of 2015 to 2018 (32 samples, 4 per period) were included. The materials analyzed were composts (14 samples), sludge (12 samples), organic fertilizers (4 samples), and manures (2 samples) from four different European countries, and they were received as a dried and finely crushed powder. The dry test samples have been shown to be stable over a number of years when stored at room temperature.

In the MARSEP programme, elements were determined as the acid-extractable form, X (ae), being *aqua regia* the most common extractant [26,27], although other extractants such as HNO_3_ or HNO_3_/H_2_O_2_ were used by a few participating laboratories. The elements considered were As, Ba, Ca, Cd, Co, Cr, Cu, Fe, Hg, K, Mg, Mn, Mo, Na, Ni, P, Pb, S, Sb, Se, Si, Sn, Sr, Ti, V, and Zn. Loss on ignition (LOI, i.e., organic matter) was also included. It was assumed that the MARSEP samples can be considered as Certified Reference Materials and that the average value for each determined element [28] obtained from the set of individual values of the participating laboratories was the “true value” for the element. The number of individual data used to calculate the average varied to a maximum of approximately 30, and depended on the element. Average values using less than *n* = 5 were not considered in this study. It is doubtful which of the two methods provided by the PXRF instrument, Soil or Mining, offers better results in relation to the determination of the majority elements (Fe, Al, Ca, K, P, S), the concentrations of which ranged in the order of percentage. There are very few cases of use of the same model of instrument for the analysis of waste samples in the literature. Havukainen et al. [23] used the Mining method, but their findings indicate that there is not an acceptable level of agreement between PXRF and *aqua regia*-ICPMS for the examined element measurements, since the differences are too large.

Two additional reference materials were used to assess the performance of the PXRF instrument for the determination of the real total content of major elements (Fe, Al, Ca, K, P, S, and Si). These samples were a sediment sample from the ISE (International Soil-Analytical Exchange) Programme (sample no. 859, period 2017.2.3, collected at De Bilt, Netherlands) [29], and sample SdAR-M2, that corresponded to a metal-rich sediment produced by the US Geological Survey [30]. For the verification check to be acceptable, it was considered that the measured value for each target analyte in these samples should be within ±20% RD of the true value [15]. The mentioned major elements could present a greater difference than trace elements between the real-total contents and the extractable contents in *aqua regia*, since they can be associated with silicate that is difficult to dissolve.

### 2.3. Statistical Data Analysis

Data were explored and transformed in the following way:

For each element, the relationships among the reported average values corresponding to MARSEP program (X (ae)) and the average reading from the SOIL (X (S)) or MINING (X (M)) methods of PXRF were assessed by plotting the data.

Multiple linear regression analysis was used to predict X (ae) as the dependent variable from X (S) or X (M) content. This first adjustment consisted of a single linear regression equation. After this first adjustment, the residuals were calculated and their correlations with other elemental contents were determined. The parameters with higher and significant Pearson coefficient (usually Si (M) but also other major and trace elements) were then added one by one into the equation, and the process was repeated to look for additional significant independent variables. X (ae) data and the predicted values obtained from the regression equations, X (predicted), were compared using a paired samples *t*-test and 99% confidence probability.

All statistical analyses were carried out with IBM^©^ SPPS^©^ Statistics version 25 (IBM Corp., Chicago, IL, USA).

The correlation coefficient (*r*) for both series of results should be 0.7 or greater for the PXRF data to be considered screening level data. If *r* is greater than 0.9 and inferential statistics (the slope of the line =1 and the y-intercept = 0) indicate the two data sets are statistically similar at the 95% confidence level, the data quality is definitive [15]. The empirical calibration (single or multiple linear regression equation) is considered quantitatively acceptable for a specific analyte if the correlation coefficient is greater than 0.98 [15].

The relative standard deviation, RSD, is the ratio as a percentage of the standard deviation, SD, and the mean concentration of an element. The relative difference, RD, is the difference, as percentage, between the predicted and actual values. When the average RD was calculated, the absolute values were used.

## 3. Results and Discussion

A summary of the results obtained for the MARSEP samples using both methods, Mining and Soil, is shown in Table 1. The range of values for several elements extended over one order of magnitude, which indicated a high variability among the different materials analyzed. These extended ranges also allowed a sufficient set of data for calibration extending between the concentration ranges that are usually given in organic amendments. For most of the elements, the number of valid results in the MARSEP programme [31] was greater than 30.

### 3.1. Loss on Ignition (LOI)

**Figure 1:**
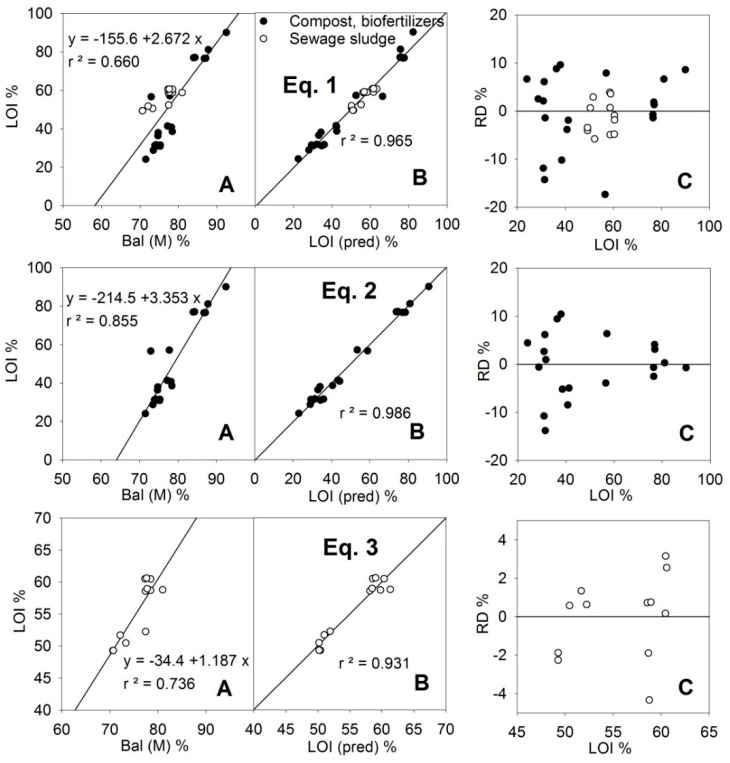
Relationships of Bal readings (difference to 100% of the sum of all measured elements) using PXRF and the LOI (loss on ignition) content in training samples of organic amendments (**A**). Adjustment of LOI predicted values using Equation (1): [LOI(pred)] = 0.998[Bal] − 0.248[Si(M)] − 0.176[Ca(M)], Equation (2): [LOI(pred)] = 1.145[Bal] − 0.272[Si(M)] − 0.268[Ca(M)] − 0.435[P(M)], and Equation (3): [LOI(pred)] = 0.885[Bal] − 0.258[Si(M)] − 0.032[Fe (M)] (Table 2) (**B**) and relative deviations (RD) of the predicted values (**C**). (*r^2^* is the coefficient of determination).

The LOI (i.e., organic matter content) is not directly determined by the PXRF instrument since the constituent elements of the organic matter (C, N, O, H) are beyond its capability. However, in the measurement method M, a result called “Bal” (balance) is given, corresponding to the difference of up to 100% that has not been assigned to other elements. Bal and LOI were correlated (Figure 1), but the adjustment was weak (*r^2^* = 0.66 for all the samples), and as can be seen in Table 1, the values for Bal were much higher than those of LOI (average data from the MARSEP programme). These two parameters adjusted well (Figure 1, Equation (1)) if the Si (M) and Ca (M) contents were included in Linear Equation (1) (Table 2) between LOI and Bal. Equation (1) assumed that Bal can include organic matter (LOI) and elements such as oxygen associated with silica, clay and calcium carbonate, that are likely constituents of the compost and sewage sludge samples. The stoichiometric coefficients in Equation (1) would be −0.114 for SiO_2_, −0.171 for SiO_3_ (silicates, clay), and −0.120 for CaCO_3_. The cause of the highest absolute values for the actual coefficients in Equation (1) is likely to be because other O-binding compounds, such as phosphates, sulfates, and iron and aluminum oxides, are included in the composition of the samples. The correlation coefficient for the predicted-LOI and actual-values, *r* = 0.982 > 0.98 (*r^2^* = 0.965), indicated the validity of the adjustment for quantification. Additionally, the slope and the y-intercept of the relationship LOI-LOI (pred) met the quality criteria (slope = 1 and y-intercept = 0) whether for Equation (1), (2) or (3).

**Figure 2:**
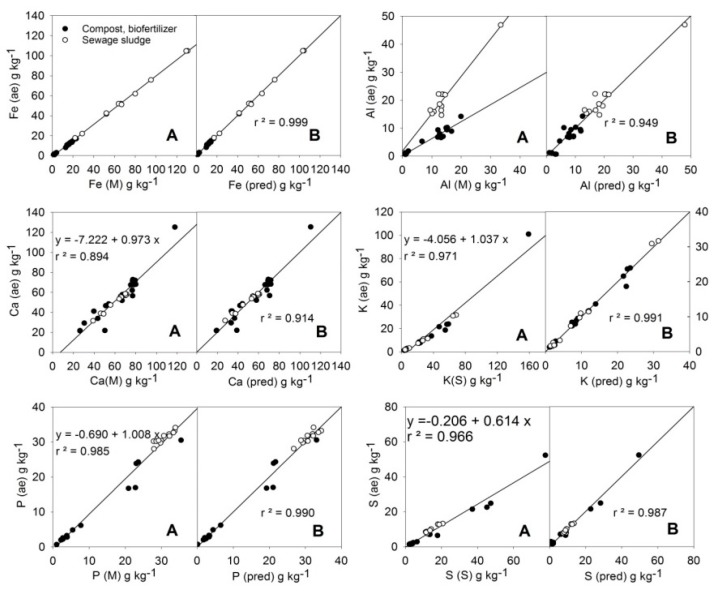
Relationships of PXRF readings of the major elements Fe, Al, Ca, K, P, and S and their *aqua regia*-extractable contents in training samples of organic amendments (**A**), and the adjustments of the predictions (**B**) of their *aqua regia*-extractable contents using the following multiple linear equations (Table 4): [Fe(pred)] = −0.242 + 0.804[Fe(M)] − 0.010[Si(M)]; [Al(pred)] = 2.077 + 1.622[Al(M)] − 0.138 [Si(M)]; [Ca(pred)] = −15.049 + 1.039[Ca(M)] + 0.145[K(S)]; [K(pred)] = −1.711 + 0.402[K(S)] + 0.117[S(S)]; [P(pred)] = −0.926 + 0.959[P(M)] + 0.033[Fe (M)]; [S(pred)] = 11.452 + 0.641[S(S)] − 0.162[Bal] + 0.004[Cu(S)].

If the samples were separated according to their type (compost or sewage sludge) an even better adjustment could be obtained for the group of compost samples using Equation (2), which included a significant P (M) term, reducing the relative error of the predictions below 15% for all the sets of samples and below 10% if the LOI value was greater than 40% (Figure 1). For the samples of sewage sludge, the obtained adjustment (Equation (3), Figure 1) included an Fe(M) term, but it did not improve on Equation (1), although since these samples had a LOI value >50% their relative errors were kept below 4%. The Fe (M) term was due to the high content of this element in sewage sludge (see Fe in Figure 2).

To the best of the authors’ knowledge, there is no previous study in which the organic matter content (LOI) of organic amendments could have been inferred directly from the PXRF data. It is worth mentioning here the works of Andrade et al. [32] and Silva et al. [33] in which they found that Ca, P, Si and Fe, in line with the results presented here, were also among the most important variables for modeling the organic matter content in Brazilian soils. However, the *r^2^* values obtained in these studies were generally <0.8, possibly due the low organic content of the Brazilian soils studied.

### 3.2. Macroelements (Fe, Al, Ca, K, P, S)

A comparison of the contents of the certified samples SdAR-M2 and ISE 859, as determined by the M and S methods of PXRF, is shown in the Table 3. The EPA Method 6200 [15] indicated that the measured value for each target analyte should be within ±20 percent (% RD) of the true value for the verification check to be acceptable. The contents of Fe (M) were satisfactory for both samples, while Fe (S) readings underestimated Fe content in the SdAR-M2 sample. The Al (M) readings underestimated its content in both samples. The Ca (M) contents were satisfactory for both samples. The Ca (S) readings were also satisfactory for both samples, although the RDs were greater than those corresponding to the M method. The K (M) content was satisfactory for the ISE 859 sample but slightly exceeded the 20% error limit for the SdAR-M2 sample. On the contrary, K (S) content was satisfactory for the SdAR-M2 sample but slightly exceeded the 20% error limit for the ISE 859 sample. Considering both methods, the average absolute RD was lower for the S method. The P (M) measurement was adequate for the ISE 859 sample, but the RD was quite high for the SdAR-M2 sample. Nevertheless, the P content of this sample (0.35 g·kg^−1^) was lower than the minimum P content in the organic amendments (1.01 g·kg^−1^, Table 1). The S (M) readings were imprecise. The content of S as read by the S method was satisfactory for the ISE 859 sample and slightly exceeded the 20% limit for the SdAR-M2 sample, but in this sample, the S content was lower than the minimum in the amendments (Table 1). The content of Si (M) was satisfactory in SdAR-M2 and slightly exceeded the 20% error limit in the ISE 859 sample. The Mg (M) content in ISE 859 was underestimated due to this element requiring air to be purged from the measurement window. In any case, the Mg element was not included in the following comparisons since a small number of data from PXRF were obtained, offering inconsistent results, possibly because they were close to the detection limit of the instrument. Additionally, in the case of Cl, there was not reported data in the MARSEP reports.

It was decided to use the readings Fe (M), Ca (M), P (M), Si (M), K (S), and S (S). The manufacturer’s recommendations indicate that the M method is especially recommended for contents of metallic elements above 1%.

Linear relationships among the average MARSEP values (X (ae)) and the selected X (M) or X (S) readings can be observed in Figure 2 for the elements Fe, Al, Ca, K, P, and S. In the case of Al, two different trends can be observed depending on the type of sample considered (compost or sewage sludge). The relationships between the average values of the MARSEP program (X (ae), the vertical axis in each plot) and the calculated values from the best equations found (X (pred), the right horizontal axis in each plot) for the majority elements are shown in Figure 2. The obtained chemical linear equation for each element is shown in Table 4, and the results of the t-test among actual and predicted *aqua regia*-extractable contents are shown in Table 5.

The linearity was excellent for Fe. However, the slope of the regression was less than 1 and the Fe (ae) results were generally 20% lower than the Fe (M) readings. Caporale et al [17] found the soil *aqua regia*-extractable content of Fe was about 10–20% lower than the measured content using a similar PXRF instrument. This indicated a fraction of Fe was highly insoluble, likely due to the crystalline structures of the clay or other silicate compounds [17], which are probably also present in the compost and sludge samples. In the case that the Si (M) content was used to adjust the linear regression, Equation (4) in Table 4 was obtained, with the coefficient for the Si(M) term being statistically significant (*p* (C) = 0.005). The correlation coefficient (*r* = 0.9999) and the results of the *t*-test (Table 5) showed a very good fitting between the actual Fe (ae) and predicted-Fe values, indicating the validity of the adjustment for quantification.

The plot of Al (M) against the corresponding MARSEP results (Al (ae)) showed two different patterns (Figure 2). Sewage sludge samples tended to have higher Al (ae) values than Mining PXRF readings, while compost and biofertilizer samples tended to give lower Al (ae) values than Mining PXRF readings. It is likely that in both amendment types, a part of their Al was associated with Si in clay structures, but the effect was different depending on the Si content of the sample (average Si (M) content was 73.1 g·kg^−1^ for the compost samples and 36.0 g kg^−1^ for the sewage sludge samples). If this factor was taken into account (Equation (5), Table 4), the coefficient obtained for the Si (M) effect was significant and only one regression line was obtained. The paired sample *t*-test (Table 5) showed no significant differences among actual and predicted Al (ae) contents, and the correlation coefficient (*r* = 0.974) was close to the limit (*r* > 0.98) to allow the quantification of this element despite the low performance of the direct Al(M) readings.

In the case of Ca, the slope of the regression line (0.973) being close to 1 (Figure 2) indicated that there is not a large fraction of Ca associated with minerals not soluble in acid. However, the dots showed a certain dispersion, which is indicative of disturbing effects, as measured by PXRF. A better fit (*r^2^* = 0.914) with significant coefficients was obtained considering K (S) contents as independent terms in the linear regression analysis (Equation (6)). The paired *t*-test (Table 5) indicated that predicted and actual Ca (ae) data did not differ at the 99% confidence interval, but the value of *r* = 0.956 < 0.98 did not allow quantification of this element. It is worth noting that the mismatch was largely due to the deviations of two points, one of them with a Ca content higher than the rest of the samples (see Figure 2). However, no anomaly was found in the general composition of these two samples that offers an explanation for their high deviations.

The slope of the regression line of K (ae) against readings of K (S) was 0.43 (Figure 2), indicating more than half of K was in acid-insoluble forms, likely associated to silicates. However, the best improvement in the adjustment was obtained if S (M) content was included in Equation (7) (Table 4). One K (ae) result >100 g·kg^−1^, corresponding to an organic fertilizer, was not used in the adjustment. With the S (M) correction the value of *r* = 0.995 indicated good quantification from the proposed equation. The paired t-test (Table 5) indicated that predicted and actual K (ae) data did not differ at the 99% confidence interval.

The fitting of the P(M) readings to the P (ae) data was good and direct (Figure 2), showing a value of slope close to 1 and *r* = 0.992 > 0.98, allowing the quantification of this element with the correction of a low value for the y-intercept. However, a further improvement can be achieved if the term Fe (M) is incorporated into Equation (8) (Table 4), reaching *r* = 0.995. The results of the *t*-test (Table 5) indicated equality among predicted and actual P (ae) data. The P–Fe relationship is likely based on the use of Fe salts used in wastewater treatment [34].

It is considered that light elements, i.e., P, K, Ca, Mg, contents are difficult to be measured with XRF, at least in soil samples [35]. In soils, Nawar et al. [35] obtained *r^2^* values of 0.92–0.95 for Mg, P, K, and Ca using the random forests model for calibration. Thus, the *r^2^* values obtained in the present study can be considered satisfactory, since the calibration procedure based on the spectra used by Nawar et al. [35] is more complex than the one used in this study.

For sulphur, the readings of the S method were about 40% higher than the acid extractable contents (Figure 2). Equation (9) provided a good fit, including the terms Bal (organic matter content) and Cu (S) content, which were both significant (Table 4). The Bal correction was negative while the Cu (S) was positive. Likely, the Cu (S) term included the effect of several metals, such as Pb, Zn, and As, which also showed correlations with the residuals (data not shown). The value *r* = 0.994 > 0.98 (Table 4) and the equality of the predicted and the actual values (Table 5) allow the quantification of S (ae).

### 3.3. Trace Elements

**Figure 3:**
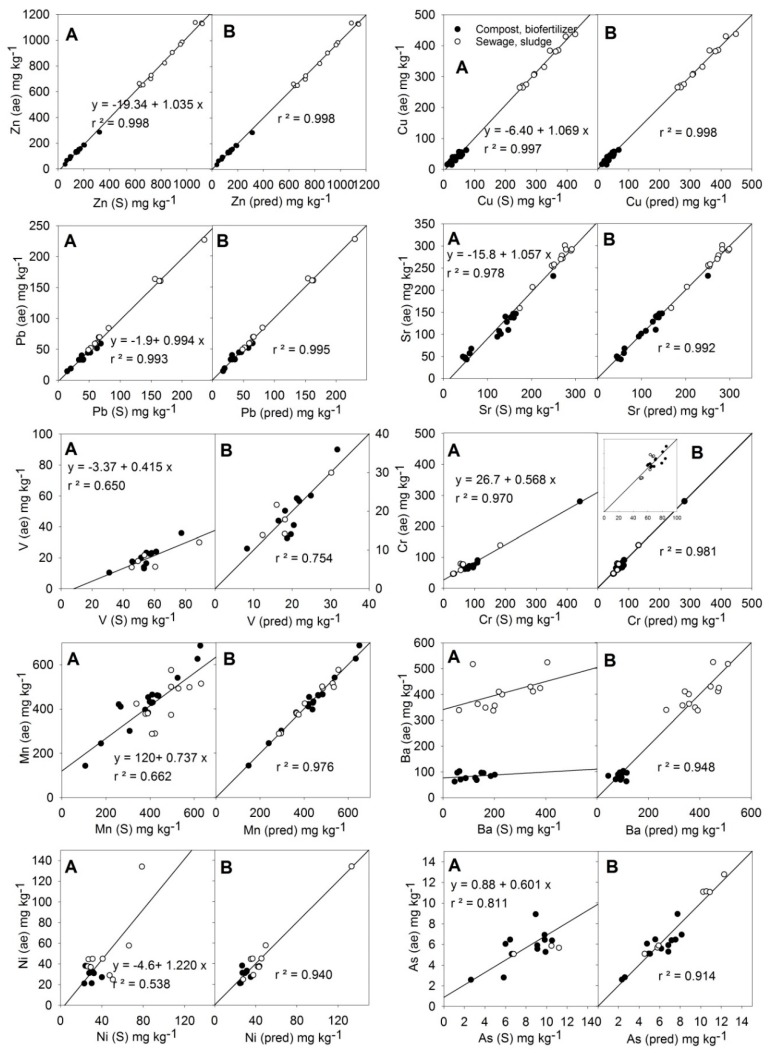
Relationships of PXRF readings of trace elements and their *aqua regia*-extractable contents in training samples of organic amendments (**A**) and the adjustments of the predictions (**B**) of their *aqua regia*-extractable contents using the multiple linear equations from Table 6.

The relationships between the average values of the MARSEP program (X (ae), vertical axis in each plot) and the values measured by the S method of PXRF (X (S), left horizontal axis in each plot) or the calculated values from the best equations found (X (pred), right horizontal axis in each plot) for the trace elements Zn, Cu, Pb, Sr, Cr, Mn, Ni, Ba, As, and V are shown in Figure 3. The obtained chemical linear equation for each element is shown in Table 6, and the result of the *t*-test among actual and predicted *aqua regia*-extractable contents is shown in Table 5.

As can be seen in Figure 3, in the case of the metals Zn, Cu, Pb, and Sr, the PXRF instrument’s direct readings quantitatively reproduced the acid-extractable contents, with *r* > 0.98 (or *r^2^* > 0.96) except *r* = 0.978 for Sr. Only minor adjustments of the y-intercept were necessary for Zn, Cu, and Sr. No other element contents improved the Zn adjustment after the correction of the y-intercept (–19.3). Equation (10) (Table 6) yielded predicted results equivalent to the actual contents (results of *t*-test, Table 5).

For Cu, Pb, and Sr, the introduction into the corresponding equations of a correction term, including the measurement of Si (M) (Equations (11)–(13), respectively, Table 6), was significant. In the case of Cu and Pb the improvement of the coefficient of determination (*r^2^*) was small because r^2^ was already close to 1 only with the single regression adjustment (Figure 3), but in the case of Sr, the adjustment (*r^2^*) improved appreciably. The *t*-test results (Table 5) and the *r* values > 0.98 (Table 6) indicated that the elements Zn, Cu, Pb, and Sr can be quantified using the given equations.

For the elements V, Cr, Mn, Ba, Ni, and As, the values measured by the S method of PXRF (X(S) did not correspond to the contents given by the MARSEP program (X (ae) (Figure 3). In the case of V, Cr, and As, the PXRF readings were greater than the MARSEP values (slope < 1). In the case of Mn and Ni, dispersion in the results was observed. In the case of Ba, compost samples and sewage sludge samples behaved differently.

Fitting Equations (14), (15), and (17) for V, Cr, and Ba, respectively, included a correction term based on Si (M) content. As was observed in relation to the majority elements, the influence of Si content probably indicated the presence of silicates and clays from soil particles in the compost and sewage samples. Vanadium could be incorporated in the mineral structures of clays, Cr occurs in soils mainly in the immobile residual fraction, and Ba may be present in some silicate mineral as impurities [36]. The fitting for V (0.7 < *r* = 0.868 < 0.9) and the t-test (Table 5) qualified the quality of the predicted data was sufficient for it to be considered as screening level data. The r for Cr (Table 6) indicated its quantification was feasible using Equation (15). Equation (17) for Ba also included a significant Al (M) term, which likely is related to aluminosilicates. In the case of Ba, r did not reach the quality criteria for quantification (*r* = 0.973 < 0.98), but the data were grouped into a single set and the level of the data quality was definitive (*r* > 0.9).

The relationship among Mn (ae) results and Mn (S) readings (Figure 2) was inadequate to allow the quantitative determination of this metal by the PXRF instrument, despite the contents of this metal being relatively high (106–631 mg·kg^−1^, Table 1). Equation (16) for Mn required several terms based on Fe (M) content, Bal (i.e., organic matter content), Ca (M), and Zr (S), which were all significant (Table 6). Manganese is a member of the iron family and both elements are closely associated with geochemical processes, and Mn oxides (as well as Fe oxides) are considered to be the most abundant compounds of the Earth’s surface, which could explain why several correction factors were required [36]. The adjustment for Mn improved very notably with Equation (16), with the prediction of quantitative quality.

Similar to the case of Mn, the relationship among the Ni (ae) results and Ni (S) readings was poor (*r^2^* = 0.538, Figure 3). The equation for Ni (Equation (18), Table 6) included the corresponding reading of the S method, and two significant terms based on Bal (organic matter) and Al (M) readings. Although the quality of predicted Ni contents should be considered definitive (*r* = 0.970, Table 6), there was a lack of dots in the range of intermediate contents (60–120 kg·kg^−1^) and additional samples will be needed to obtain a better chemical regression equation.

Equation (19) for As (Table 6) included a Ca (M) term, and allowed us to obtain definitive data (r = 0.921 > 0.9) for this element despite its low concentrations (average As (S) = 9.8 mg·kg^−1^, Table 1). The guideline detection limit given by the manufacturer for this element is 10 mg·kg^−1^ [37], so many of the readings obtained only slightly exceeded this limit.

### 3.4. Deviations of Results

**Figure 4:**
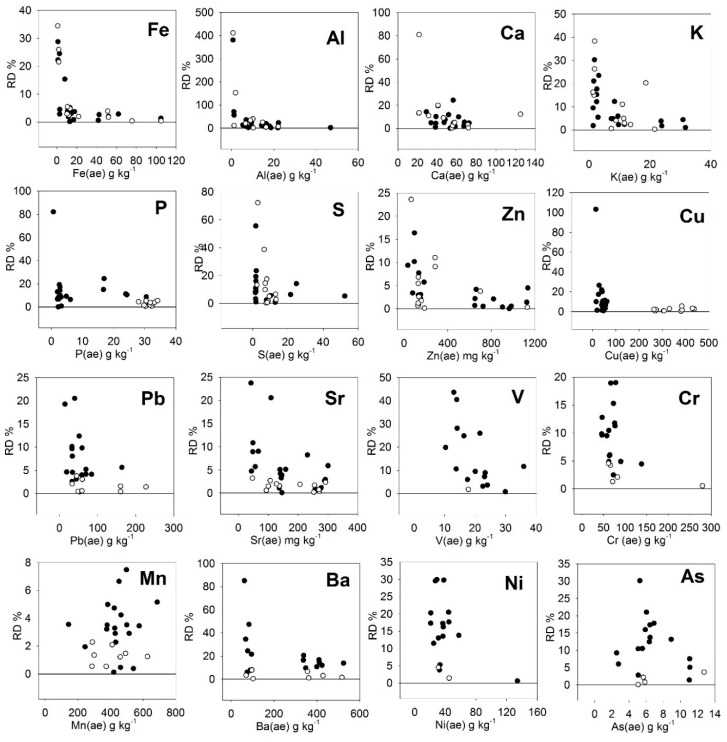
Absolute values of the relative deviations (RD) among predicted and actual *aqua regia*-extractable contents for major and trace elements.

The absolute values of the relative deviations (RD) among predicted and actual *aqua regia*-extractable contents for the considered elements are shown in Figure 4. The information from Figure 4 is summarized in Table 7. The average RD was very high (48%) for Al, and then followed the order Ba, V, Ni, S, and K, being less than 10% for the rest of the elements. Consequently, for the elements Al, Ba, V, Ni, S, and K there were 13, 6, 5, 5, 4, and 6 results surpassing the 20% RD (Table 7). Nevertheless, as can be observed in Figure 4, for all the elements there was a sharply decay in the RD as their contents increased, and this 20% limit was exceeded only in the case of samples with the lowest concentrations of the elements considered. Hence, if the data with an RD greater than 20% are not included, the average RD was less than 10% for all the elements except Ni (last column in Table 7). The calculated content above which the RD will be lower than 20% (content for RSD < 20%) is also shown in Table 7.

The content for RSD < 20% was lower than the limits for macroelements or trace metals given in the EU regulations for organic fertilizers [38] (Table 7), which means that the PXRF technique could be valid to test the compliance of this type of product, at least in terms of quality control to be performed in the field or in the factory.

### 3.5. Practical Implications of this Study

The results of this study clearly indicate that rapid and accurate analysis of organic amendments using XRF instruments is possible. Considering that these types of analysis are high in cost, time consuming, and use disposable laboratory ware and potentially hazardous reagents used in the conventional laboratory methods for the characterization (nutrients, trace elements, and organic matter content), the proposed alternative would be not only viable but also recommended. Above all, the XRF analytical approach proposed would be very useful for quality control tasks in waste treatment facilities (composting plants, sewage treatment plants), organic fertilizer factories, nursery growing media, and for field control in small farms scale, i.e., urban agriculture or experimental or research trials. In these situations, we believe that the XRF technique represents a valuable decision support tool aimed at the proper management of farms/factories.

The calibration equations obtained in this study were obtained with well characterised set of samples from composts, manure, sewage sludge, and biofertilizers from four countries in Central Europe. The applicability of the equations should be checked with other types of organic samples. Continuous and local or regional calibrations are also recommended to reinforce and generalize the relationships found or to find specifically-fitted ones. Interlaboratory trials are also desirable in order to adapt and improve the resolution of the technique for specific organic matrices and the cross-check of equipment from different brands and suppliers.

The XRF equipment used did not reach an adequate detection limit for elements such as Cd and Hg that are of interest in organic amendments. It would be necessary to look for calibrations to infer the contents of these elements. Such calibrations could be specific in terms of geographical area or sample type. It would also be useful to find calibrations allowing to infer other relevant properties for the characterization of amendments or organic fertilizers, such as electrical conductivity, as has been already done in soils [39]. Future work is also envisaged to investigate the potential combination of data from XRF with that obtained using other techniques like near- and mid-infrared spectroscopy that may provide complementary information to infer other variables relative to the composition or particular properties of organic amendments.

## 4. Conclusions

The results of this study have shown that in samples of organic amendments (composts, manures, biofertilizers, and sewage sludge), it is possible to determine the contents of *aqua regia*-soluble elements from the measurements made with a portable XRF analyzer. It was also possible to determine the content of organic matter (LOI) which is a novel achievement for this kind of analysers. The analyses were based on multiple linear equations that corrected the readings obtained for each element with the readings obtained from certain majority elements, such as Si, Fe, Al, Ca, K, or S. For LOI and the elements Fe, K, P, S, Zn, Cu, Pb, Sr, Cr, and Mn, the formulas used gave results of quantitative quality, while for Al, Ca, V, Ba, Ni, and As, the results can be considered definitive. The correction factors we used indicated that the lineal transformations found are based on the chemical composition of the sample matrix. Although this study was carried out with dried and ground samples, the speed of the scanning required for the analysis (3.5 min in total) would allow its application to heterogeneous dried raw samples by performing several repetitions in different aliquots of the same sample. Given that the correction factors only make use of the PXRF analyzer readings and no other laboratory parameters are required, the proposed method can be considered as autonomous and fully usable in field conditions. The PXRF instruments are achieving significant improvements in the analysis of numerous soil sample parameters, but their applicability and reliability for organic amendments were, until now, limited. This study highlights the potential of XRF portable analyzers for fast, cost-effective and reliable analysis of nutrients and heavy metals in organic amendments. The high coefficients of determination found for the predictions exceed those of many other applications in other fields. This study extends the fields of application of the XRF technique to the routine analysis of organic amendments.

## Figures and Tables

**Table 1:** Comparison of ranges of elemental concentrations (mg kg^−1^ unless otherwise stated) determined using portable X-ray spectrometry (PXRF).

Samples	Method Soil				Method Mining			
	N ^1^	Min	Max	Mean	N ^1^	Min	Max	Mean
LOI ^2^	32	24.1	90.0	52.0				
Bal ^3^					32	70.6	92.5	77.7
As	20	2.6	18.5	9.8	14	4.7	10.0	7.2
Ba	23	45.4	406	177	24	79.5	489	244
Cr	21	30.4	441	94.0	25	92.6	326	170
Cu	31	12.0	426	150.6	27	29.2	768	294
Mn	28	106	631	418	27	105	680	312
Mo	23	3.2	22.7	5.2	10	2.9	16.2	5.0
Ni	18	23.0	78.6	36.8		<LOD	<LOD	<LOD
Pb	25	14.3	236	69.8	24	12.9	240	66.3
Rb	32	8.7	59.9	26.8	32	4.3	38.0	17.2
Sr	32	44.2	292	170	32	44.6	296	172
Ti	32	68.5	4142	1664	32	97.7	2669	1200
V	16	31.0	88.8	56.0	24	42.5	157	82.7
Zn	32	55.5	1122	419	32	59.0	1364	502
Zr	32	4.46	128	59.3	32	3.8	120	51.7
Al ^4^					30	1.0	33.5	11.4
Ca ^4^	32	33.6	145	79.2	32	26.4	118	61.0
Cl ^4^					32	0.30	10.3	2.57
Fe ^4^	32	0.9	147	29.9	32	1.4	130	33.3
K ^4^	32	3.74	159	28.5	32	2.56	94.9	15.9
Mg ^4^					12	3.10	7.19	4.33
P ^4^					32	1.01	35.4	17.1
S ^4^	32	2.22	77.6	14.6	32	3.64	92.8	18.0
Si ^4^					32	4.08	159	59.2

^1^ N, valid cases above limit of detection (LOD). ^2^ LOI: Loss on ignition values in %. Data from the MARSEP programme, not from PXRF measurements. ^3^ Bal: Difference to 100% of the sum of all measured elements. ^4^ Values in g kg^−1^.

**Table 2:** Multiple linear equations for the prediction of LOI ^1^ from the Bal ^2^ readings obtained by the PXRF using the Mining (M) method.

Equation	[X(pred)]=A [W(M)]+B [X(M)]+C [Y(M)]+D [Z(M)]	*p* Values				Paired *t*-test		
		*p*(A)	*p*(B)	*p*(C)	*p*(D)	*r*	Mean	Sd ^4^
(1) ^2,3^	[LOI(pred)]=0.998 [Bal]−0.248 [Si(M)] −0.176[Ca(M)]	0.000	0.000	0.000		0.982	52.0	3.31
(2)	[LOI(pred)]=1.145 [Bal]−0.272 [Si(M)]−0.268[Ca(M)]−0.435[P(M)]	0.000	0.000	0.000	0.000	0.993	49.8	2.53
(3)	[LOI(pred)]=0.885 [Bal]−0.258 [Si(M)]−0.032[Fe(M)]	0.000	0.000	0.019		0.965	55.8	1.24

^1^ LOI: Loss on ignition, i.e., organic matter content. Values in %. ^2^ [Bal]: Difference to 100% of the sum of all measured elements. ^3^ Si (M), Ca(M) and P(M) results expressed in g kg^−1^. ^4^ sd, standard deviation of the differences.

**Table 3:** Real total contents (g kg^−1^) of the sediment samples ISE 859 and SdAR-M2 as determined using the Mining and Soil methods of the portable X-ray spectrometer (PXRF).

		Cert ^1^		Mining			Soil	
		Value	Value	sd ^2^	RD(%) ^3^	Value	sd ^2^	RD(%) ^3^
Fe	ISE 859	42.0	46.4	1.9	10.6	37.7	0.1	−10.2
	SdAR_M2	18.4	18.5	0.1	0.3	13.9	0.1	−24.3
Al	ISE 859	55.3	37.5	0.1	−32.2	-	-	-
	SdAR_M2	66.0	46.8	0.5	−29.0	-	-	-
Ca	ISE 859	33.9	35.0	0.0	3.1	39.5	0.2	16.6
	SdAR_M2	6.00	5.72	0.1	−4.7	5.45	0.0	-8.7
K	ISE 859	15.3	12.3	0.0	−19.2	18.5	0.0	21.5
	SdAR_M2	41.5	32.7	0.3	−21.2	40.1	0.2	-8.7
P	ISE 859	4.08	4.09	0.03	0.3	-	-	-
	SdAR_M2	0.35	0.58	0.11	68.3	-	-	-
S	ISE 859	11.7	17.9	0.1	53.4	11.5	0.5	−1.1
	SdAR_M2	0.97	1.56	0.04	60.6	1.19	0.08	22.6
Si	ISE 859	213.0	167.5	0.3	−21.3	-	-	-
	SdAR_M2	343.3	304.5	1.1	−11.3	-	-	-
Mg	ISE 859	8.42	5.42	0.45	−35.7	-	-	-
	SdAR_M2	2.95	-	-	-	-	-	-

^1^ Certified value. ^2^ sd, standard deviation. ^3^ RD, percentage of difference to the certified value.

**Table 4:** Multiple linear equations for the prediction of *aqua regia* extractable contents of majority metal (X (pred)) from the readings obtained by the PXRF.

Equation ^1^	[X(pred)]=A B [W(M,S)]+C [X(M,S)]+D [Y(M,S)]+E [Z(M,S)]	*p* Values					
		*p*(B)	*p*(C)	*p*(D)	*r*	Mean ^2^	N ^4^
(4)	[Fe(pred)]=−0.242+0.804 [Fe(M)]−0.010 [Si(M)]	0.000	0.005		1.000	25.9	32
(5)	[Al(pred)]=2.077+1.622 [Al(M)]−0.138 [Si(M)]	0.000	0.000		0.974	12.0	30
(6)	[Ca(pred)]=−15.049+1.039 [Ca(M)]+0.145 [K(S)]	0.000	0.017		0.956	52.1	32
(7)	[K(pred)]=−1.711+0.402 [K(S)]+0.117 [S(S)]	0.000	0.000	0.000	0.995	9.53	31
(8)	[P(pred)]=−0.926+0.959 [P(M)]+0.033[Fe(M)]	0.000	0.001		0.995	16.6	32
(9) ^3^	[S(pred)]=11.452+0.641[S(S)]−0.162[Bal] +0.004[Cu(S)]	0.000	0.001	0.011	0.994	8.30	31

^1^ Readings from M or S method. ^2^ Values in g kg^−1^ except Cu(S) in mg kg^−1^ and Bal in %. ^3^ Bal: Difference to 100% of the sum of all measured elements. ^4^ N, number of data points.

**Table 5:** Paired samples t-test between actual and predicted *aqua regia*-extractable elemental contents using the multiple regression equations.

	Paired Differences			99% CID ^3^				
	Mean	Sd ^1^	sEM ^2^	Lower	Upper	t	Df ^4^	Sig ^5^
Fe	0.000	0.77	0.14	−0.37	0.37	0.000	31	1.000
Al	0.000	2.17	0.40	−1.09	1.09	0.000	29	1.000
Ca	0.000	6.52	1.15	−3.16	3.16	0.000	31	1.000
K	0.000	1.34	0.24	−0.66	0.66	0.000	30	1.000
P	0.000	1.37	0.24	−0.67	0.67	0.000	31	1.000
S	0.000	1.13	0.20	−0.56	0.56	0.000	30	1.000
Zn	0.000	0.77	0.14	−0.37	0.37	0.000	31	1.000
Cu	0.000	7.53	1.30	−3.58	3.58	0.000	30	1.000
Pb	0.000	3.72	0.74	−2.08	2.08	0.000	24	1.000
Sr	0.000	7.74	1.37	−3.76	3.76	0.000	31	1.000
V	0.000	3.32	0.83	−2.45	2.45	0.000	15	1.000
Cr	0.000	6.82	1.49	−4.24	4.24	0.000	20	1.000
Mn	0.000	17.7	3.35	−9.27	9.27	0.000	27	1.000
Ba	0.000	39.0	8.13	−22.9	22.9	0.000	22	1.000
Ni	0.000	6.14	1.44	−4.20	4.20	0.000	17	1.000
As	0.000	1.08	0.24	−0.69	0.69	0.000	19	1.000

^1^ sd, standard deviation of the differences. ^2^ sEM, standard error of the mean of the differences. ^3^ CID, 99% Confidence Interval of the Difference. ^4^ df, degrees of freedom. ^5^ sig, two-tailed significance level.

**Table 6:** Linear equations for the prediction of *aqua regia* extractable contents of trace metal (X(pred) from the readings obtained by the PXRF.

Equation ^1^	[X(pred)]=A+ B [W(S)]+C [X(M,S)]+D [Y(M,S)] +…	*p* Values					
		*p*(B)	*p*(C)	*p*(D)	*r*	Mean ^2^	N ^4^
(10)	[Zn(pred)]=−19.34+1.035 [Zn(S)]	0.000			0.999	414.7	32
(11)	[Cu(pred)]=1.69+1.057 [Cu(S)]−0.103[Si(M)]	0.000	0.004		0.999	154.6	31
(12)	[Pb(pred)]=3.48+0.977 [Pb(S)]−0.059[Si(M)]	0.000	0.005		0.998	67.5	25
(13)	[Sr(pred)]=−1.65+1.055 [Cu(S)]−0.231[Si(M)]	0.000	0.000		0.996	164.0	32
(14)	[V(pred)]=−7.09+0.377 [V(S)]−0.060[Si(M)]	0.000	0.035		0.868	19.8	16
(15)	[Cr(pred)]=36.68+0.560 [Cr(S)]−0.117[Si(M)]	0.000	0.005		0.991	80.1	21
(16)	[Mn(pred)]=−506.1+0.847 [Mn(S)]−0.928[Fe(M)]+8.118 [Bal]−1.087[Ca(M)]+0.975[Zr(S)]	0.000 0.000	0.000 0.000	0.000	0.988	428.2	28
(17)	[Ba(pred)]=183.6+0.583 [Ba(S)]−3.045[Si(M)]+13.25 [Al(M)]	0.000	0.000	0.000	0.973	250.9	23
(18)	[Ni(pred)]=−397.9+0.949[Ni(S)]+4.917[Bal] +2.260[Al(M)]	0.000	0.000	0.000	0.970	40.3	18
(19) ^3^	[As(pred)]=3.857+0.525 [As(S)]−0.034[Ca(M)]	0.000	0.058 ^ns^		0.921	6.80	20

^1^ Readings from M or S method depending on the elelment. ^2^ Values in mg kg^−1^ except Si (M), Fe (M), Ca (M) and Al (M) in g kg^−1^ and Bal in %. ^3^ Bal: Difference to 100% of the sum of all measured elements. ^4^ N, number of data points. ^ns^ no significative.

**Table 7:** Deviations between actual and predicted contents.

	Rsd (%) ^1^	Content for RSD < 20% ^2^	EU Limit	Actual Values		
				*n* > 20% ^3^	Average RD (%)	Average RD-*n*(%) ^4^
LOI ^5^	6.4	16.6	27 ^7^	0	5.1	5.1
Fe ^5^	3.0	3.9		6	7.4	3.1
Al ^5^	18.1	10.9		13	47.9	7.5
Ca ^5^	12.5	32.6		2	9.7	6.9
K ^5^	14.1	6.7	16.6 ^7^	6	10.4	6.5
P ^5^	8.3	6.9	8.7 ^7^	2	9.8	6.9
S ^5^	13.6	5.7		4	12.5	7.3
Zn ^6^	0.18	3.9	800 ^8^	1	4.5	3.9
Cu ^6^	4.9	37.7	300 ^8^	3	9.8	5.5
Pb ^6^	5.5	18.6	120 ^8^	1	5.7	5.0
Sr ^6^	4.7	38.7		2	4.6	3.4
V ^6^	16.8	16.6		5	15.4	7.6
Cr ^6^	8.5	34.1		0	8.1	8.1
Mn ^6^	4.1	88.5		0	3.1	3.1
Ba ^6^	15.5	195		6	16.2	8.2
Ni ^6^	15.2	30.7	50 ^8^	5	14.8	10.5
As ^6^	15.9	5.4	40 ^8^	2	10.0	8.3

^1^ RSD, relative standard deviation of the differences = sd (Table 5) × 100 / mean MARSEP value. ^2^ Content for RSD < 20%. Limit of content above which RD will be lower than 20% = 5 × sd (Table 5). ^3^ *n* > 20%, number of results with RD > 20%. ^4^ Average RD-n, average RD if the results with RD > 20% are eliminated. ^5^ In g kg^−1^. ^6^ In mg kg^−1^. ^7^ Minimum requirement for organic fertiliser type following [33]. ^8^ Maximum allowable limit for organic fertiliser type following [33].

## Abbreviations

ICPMS	Inductively coupled plasma—Mass Spectrometry
ICP-OES	Inductively coupled plasma—Optical emission spectrometry
ISE	International Soil Exchange
LOI	Loss on ignition
MARSEP	Manure and Refuse Sample Exchange Programme
PXRF	Portable X-ray fluorescence
RD	Relative difference: difference, as percentage, between the predicted and actual values
RSD	Relative standard deviation
